# Activation of ASIC3/ERK pathway by paeoniflorin improves intestinal fluid metabolism and visceral sensitivity in slow transit constipated rats

**DOI:** 10.1002/kjm2.12829

**Published:** 2024-04-17

**Authors:** Yuan Deng, Qiong Zhao, Hong‐Yun Zhou, Zi‐Qi Zhang, Yu Zhan

**Affiliations:** ^1^ Department of Chinese Pediatrics College of Clinical Medicine, Chengdu University of Traditional Chinese Medicine Chengdu China; ^2^ Department of Pediatrics Hospital of Chengdu University of Traditional Chinese Medicine Chengdu China; ^3^ Department of Anorectal Chengdu First People's Hospital Chengdu China

**Keywords:** acid‐sensitive ion channel 3/extracellular signal‐regulated kinase signaling pathway, fluid metabolism, paeoniflorin, slow transit constipation, visceral sensitivity

## Abstract

Slow transit constipation (STC) is one of the most common gastrointestinal disorders in children and adults worldwide. Paeoniflorin (PF), a monoterpene glycoside compound extracted from the dried root of *Paeonia lactiflora*, has been found to alleviate STC, but the mechanisms of its effect remain unclear. The present study aimed to investigate the effects and mechanisms of PF on intestinal fluid metabolism and visceral sensitization in rats with compound diphenoxylate‐induced STC. Based on the evaluation of the laxative effect, the abdominal withdrawal reflex test, enzyme‐linked immunosorbent assay, quantitative real‐time polymerase chain reaction, western blot, and immunohistochemistry were used to detect the visceral sensitivity, fluid metabolism‐related proteins, and acid‐sensitive ion channel 3/extracellular signal‐regulated kinase (ASIC3/ERK) pathway‐related molecules. PF treatment not only attenuated compound diphenoxylate‐induced constipation symptoms and colonic pathological damage in rats but also ameliorated colonic fluid metabolic disorders and visceral sensitization abnormalities, as manifested by increased colonic goblet cell counts and mucin2 protein expression, decreased aquaporin3 protein expression, improved abdominal withdrawal reflex scores, reduced visceral pain threshold, upregulated serum 5‐hydroxytryptamine, and downregulated vasoactive intestinal peptide levels. Furthermore, PF activated the colonic ASIC3/ERK pathway in STC rats, and ASIC3 inhibition partially counteracted PF's modulatory effects on intestinal fluid and visceral sensation. In conclusion, PF alleviated impaired intestinal fluid metabolism and abnormal visceral sensitization in STC rats and thus relieved their symptoms through activation of the ASIC3/ERK pathway.

## 1 INTRODUCTION

Slow transit constipation (STC) is a chronic disease characterized by decreased or absent colonic peristalsis and delayed colon emptying; it is the primary type of functional constipation (FC).^1^ The global prevalence of FC in children and adults is 9.5% and 14%, respectively.^2^ Because of its long duration and recurrent symptoms, FC seriously affects patients' physical and mental health and quality of life.^3^ Currently, STC treatment is based on dietary guidance, education, and behavioral therapy, combined with laxatives and probiotics, but their long‐term efficacy is poor.^2^ Frequent use of laxatives has pronounced side effects and is also prone to dependence, making treatment more difficult.^4^ Therefore, exploring new strategies and targets for safe and effective STC treatment is urgently needed.

Paeoniflorin (PF) is the main active ingredient extracted from the dried root of *Paeonia lactiflora* from the family Ranunculaceae.^5^ It possesses various pharmacological attributes, including anti‐inflammatory, immunomodulatory, anti‐tumor, and anti‐depressant uses.^5^, ^6^ Previous studies demonstrated that PF can treat constipation by activating the PLC‐γ1/PIP2 pathway and TRPA1 channel to promote 5‐hydroxytryptamine (5‐HT) release from enterochromaffin cells.^7^ Although the efficacy of PF on STC has been established, its mechanisms of action have yet to be fully elucidated. This is partly due to the complex nature of the pathogenesis of STC and the multi‐targeted action of natural drugs. Therefore, the laxative mechanism of PF requires further investigation. Decreased intestinal fluid and reduced rectal sensitivity can lead to fecal dryness and delayed colonic emptying, which are essential causes of constipation.^8^, ^9^ Aquaporins (AQPs) mediating intestinal luminal water transfer and mucins (MUCs) controlling intestinal mucus secretion are related to intestinal fluid metabolism.^8^, ^10^ The excitatory neurotransmitter 5‐HT and the inhibitory neurotransmitter vasoactive intestinal peptide (VIP) are associated with visceral sensitization.^11^, ^12^ However, it is uncertain whether PF regulates liquid metabolism protein expression and visceral sensitization‐related neurotransmitter secretion in STC rats to produce a laxative effect.

Acid‐sensitive ion channel 3 (ASIC3), a protein that can be activated by an extracellular acidifying environment, is abundantly expressed in the colon and involved in regulating gastrointestinal motility and bladder emptying.^13^ Inhibiting ASIC3 has improved visceral hypersensitivity and gastrointestinal function in post‐infectious irritable bowel syndrome (IBS) rats.^14^ It was discovered that in wild‐type mouse fibroblast‐like synoviocytes, both acidic and inflammatory conditions activated ASIC3 expression; however, extracellular signal‐regulated kinase phosphorylation (p‐ERK) was decreased when only acidic conditions were present, whereas p‐ERK was increased when both acidic and inflammatory conditions were present, leading to cell death and inhibition of synovial proliferation.^15^ Thus, ASIC3 can regulate p‐ERK, but its regulatory effect varies under different conditions. Activating ERK not only plays a role in treating STC by Xiao‐Cheng‐Qi‐Tang, a combination of three herbs, but may also be involved in regulating AQPs and visceral sensitization in IBS.^16^, ^17^ PF may exert neuroprotective effects by regulating ASIC's activity and protein expression in rat pheochromocytoma cells.^18^ In addition, PF also attenuated the neurological damage in the depression model by activating the ERK‐CREB signaling pathway in hippocampal neurons.^19^ In other words, PF can regulate ASICs and ERK in neurological disorders. The above studies have demonstrated the essential role of ASIC3 and ERK in intestinal diseases. Additionally, the relationship between ASIC3 and ERK regulation and the effects of PF on ASICs and ERK have been observed in other systemic diseases. However, it remains to be investigated whether PF may modulate gut fluid and visceral sensation in STC rats by regulating the ASIC3/ERK pathway.

The present study aimed to investigate the effects and mechanisms of PF on compound diphenoxylate (CDiph)‐induced colonic fluid and gut sensation in rats. We hypothesized that PF could ameliorate intestinal fluid dysmetabolism and abnormal visceral sensitization by activating the ASIC3/ERK pathway, thus alleviating constipation symptoms in STC rats.

## 2 MATERIALS AND METHODS

### 2.1 Animals

Sixty 4‐week‐old SPF Sprague–Dawley rats (60–80 g), half male and half female, were purchased from SiPeiFu Biotechnology Co., Ltd (Beijing, China). They were housed in a laboratory with 55 ± 10% humidity, 25 ± 2°C temperature, 12/12 h alternating light/dark, and fed ad libitum. All experimental procedures were approved by the Ethics Committee of the Sichuan Laboratory Animal Society (approval number: P202303201).

### 2.2 Animal treatments

Rats were randomly divided into five groups (*n* = 12, half male and half female) after 1 week of acclimation feeding: control group (control), model group (STC), lactulose group (STC + LOS), PF group (STC + PF), and amiloride group (STC + PF + Amiloride). The STC model was induced by the reference method,^20^ but the dose was adjusted. The control group was gavaged with saline, and the other groups were gavaged with CDiph (15 mg/kg, H45020028, Guangxi Hefeng Pharmaceutical Co., Ltd, Hechi, China) once a day for 14 consecutive days. After the model was established, the control and STC groups were gavaged with saline, the LOS group was gavaged with lactulose oral solution (3.5 mL/kg, Abbott Biologicals B.V. Olst, The Netherlands), and the PF group was gavaged with PF (40 mg/kg, A0133, Chengdu must bio‐technology Co., Ltd, Chengdu, China). The amiloride group was gavaged with PF and injected intraperitoneally with amiloride hydrochloride (20 μg/kg, HY‐B0285A, Haoyuan Chemexpress Co., Ltd, Shanghai, China), while the other groups were simultaneously injected intraperitoneally with saline. The above treatments were administered once daily for 14 consecutive days. At the end of treatment, the rats were anesthetized, and blood collected by abdominal aortic puncture. Serum and colon tissues were collected and preserved for examination.

### 2.3 Defecation experiment

Rat feces were collected individually 24 h after treatment completion, counted, and scored using Bristol fecal typing criteria^21^ (Table 1). Feces were measured in wet weight (WW), then dried at a constant temperature of 60°C for 12 h and weighed as dry weight (DW). The water content of the feces was calculated as follows^7^: [(WW − DW)/WW] × 100%.

**TABLE 1:** Fecal Bristol Scale.

Grade (score)	Fecal character
Grade 1 (1 point)	Dispersed hard lumps resembling nuts
Grade 2 (2 points)	Sausage‐like, but in chunks
Grade 3 (3 points)	Sausage‐like, but with surface cracks
Grade 4 (4 points)	Sausage‐ or snake‐like, smooth and soft
Grade 5 (5 points)	Soft mass with clear margins
Grade 6 (6 points)	Fluffy, poorly defined, mushy stools
Grade 7 (7 points)	Watery, no solids

### 2.4 Evaluation of visceral sensitivity

Visceral sensitivity was evaluated using the abdominal withdrawal reflex (AWR) score using the colorectal dilatation method.^21^ Rats were anesthetized with ether (LOT: 2204017, Xilong Scientific Co., Ltd, Shantou, China) after 12 h of fasting. An 8F catheter sheathed with a 6 cm latex finger cuff (balloon), a sphygmomanometer, and a pressurized device were connected via a three‐way valve. The balloon was inserted about 5 cm into the anus of the rats, followed by fixing the catheter at the anus. Finally, the rats were placed in a transparent cage. After the rats were awakened and acclimated to the environment, air was uniformly injected into the balloon. Subsequently, the AWR score was determined by maintaining the pressure at 20, 40, 60, and 80 mmHg for 30 s each. The visceral pain threshold (defined as a pressure value of AWR = 3) was recorded. The test was repeated three times with 10‐min intervals between each trial, with the average value of the three trials taken as the final result. Criteria for assessing AWR scores can be found in Table 2.

**TABLE 2:** AWR scoring criteria.

Score	Behavior of rats
0	No apparent changes in rats
1	Rats were emotionally unstable and remained still, with occasional twisting or up‐and‐down movements of the head.
2	Slightly retracted abdominal muscles, but abdominal muscles did not lift off the bottom of the cage.
3	Rats' abdominal muscles contract more strongly and lift off the bottom of the cage.
4	Rats' abdominal muscles are strongly contracted, the abdomen is bowed, the perineum ultimately leaves the bottom of the cage, and the pelvis is lifted.

Abbreviation: AWR, abdominal withdrawal reflex.

### 2.5 Intestinal transport experiment

The rats were gavaged with 10% activated charcoal suspension (gavage dose: 10 mL/kg, materials purchased from Shanghai Macklin Biochemical Technology Co., Ltd, China.) before sampling and executed after 30 min.^7^ Small intestinal tissue was taken. The total length of the small intestine (L1) and the length of activated charcoal propelled in the intestinal canal (L2) were measured. The small intestine propulsion rate was calculated as (L2/L1) × 100%.

### 2.6 Histopathological testing

Colon tissues were fixed in 4% paraformaldehyde (BL539A, Biosharp, Hefei, China) for 24 h before embedding in paraffin. Sections were stained with hematoxylin and eosin (BL700B, Biosharp, Hefei, China), and lesions were observed with a microscope (DM500, Leica). CaseViewer 2.4 (3DHISTECH, Hungary) software was used to measure and take pictures.

### 2.7 Quantitative real‐time polymerase chain reaction analysis

Colon tissue RNA was extracted with Trizol (R0016, Beyotime Biotechnology, Shanghai, China) and DEPC H_2_O (BL510B, Biosharp, Hefei, China). Reverse transcription and amplification were successively performed with the reverse transcription kit 2× RT OR‐Easy TM Mix (RT‐01022, Foregene Co., Ltd, Chengdu, China) and the amplification kit Fast SYBR Green qPCR Master Mix UDC (A402, Exongen Biotechnology Co., Ltd, Chengdu, China), both according to manufacturers' instructions. The CT values were statistically analyzed using the delta–delta equation (2^−ΔΔCT^), and the data of model groups were used for homogenization. The primer information is shown in Table 3.

**TABLE 3:** Primer sequences for qPCR.

Gene	Forward primer(5′–3′)	Reverse primer(5′–3′)
ASIC3	GCTCCTCTTCCAAATCCTG	CTCAAACGGGGTCTCTTCT
ERK1	CCACAAAACTTGACAGACCA	CAGAGAAGGAGCAGGTAGGA
ERK2	ACACGCCTTTCCTTGATTT	CACATCTTTCTTGGCATTTCT

### 2.8 Enzyme‐linked immunosorbent assay (ELISA)

Colon tissues were homogenized in phosphate buffer saline (ZLI‐9062, ZSGB‐BIO, Beijing, China) at a mass‐to‐volume ratio of 1:9. The supernatant was collected after centrifugation (*G* = 5000 *g* for 10 min), and the protein concentration was detected by BCA kit (AR0146, Boster, Wuhan, China). The total concentration was adjusted to 2.62 mg/mL. Frozen serum samples were assayed after natural recovery to room temperature. Rat MUC2 ELISA KIT (ZC‐37588), Rat AQP‐3 ELISA KIT (ZC‐37215), Rat VIP ELISA KIT (ZC‐56352), and Rat 5‐HT ELISA KIT (ZC‐35959‐J), all purchased from ZCIBIO Technology Co., Ltd (Shanghai China), were used to detect the expression levels of MUC2 and AQP3 in colon tissues and the levels of serum 5‐HT and VIP, respectively.

### 2.9 Immunohistochemical assay

Paraffin sections of colon tissue were deparaffinized and hydrated, then sequentially subjected to antigen repair, endogenous peroxidase blockade, and BSA (GC305010, Servicebio, Wuhan, China) closure. They were then incubated with ASIC3 antibody (1:100, ab302776, Abcam, Cambridge, England) at 4°C overnight, and then with HRP sheep anti‐rabbit IgG (1:100, GB23303, Servicebio, Wuhan, China) at 37°C for 30 min. The color was developed with a DAB kit (ZLI‐9018, ZSGB‐BIO, Beijing, China), and the nuclei were restained with hematoxylin (LM10N13, J&K Scientific, Beijing, China). Sections were observed with a microscope (BA410, Motic) and analyzed with CaseViewer 2.4 after image acquisition.

### 2.10 Western blot (WB)

The total protein of colon tissue was extracted, and protein concentration was measured by BCA kit (AR0146, Boster, Wuhan, China). The denatured protein samples were separated on SDS‐PAGE, transferred to a PVDF membrane (IPVH00010, Merck Millipore, Darmstadt, Germany), and blocked with 5% BSA (GC305010, Servicebio, Wuhan, China). The membrane was incubated with antibodies MUC2 (1:5000, A4767, ABclonal, Wuhan, China), AQP3 (1:1000, BA1559, Boster, Wuhan, China), ASIC3 (1:500, BA2745‐2, Boster, Wuhan, China), ERK1/2 (1:1000, T40071, Abmart, Shanghai, China), p‐ERK1/2 (1:1000, TA1015, Abmart, Shanghai, China), and β‐actin (1:5000, GB11001, Servicebio, Wuhan, China) at 4°C overnight, and then incubated with HRP sheep anti‐rabbit IgG + HRP sheep anti‐mouse IgG (1:2000, BA1056, Boster, Wuhan, China) for 1 hour at room temperature. After spiking the PVDF membrane with an ECL developer (4AW011, 4ABiotech, Suzhou, China), the protein band images were captured and analyzed using a chemiluminescence developer (Touch Imager Pro, e‐BLOT, China) and ImageJ software, respectively.

### 2.11 Statistical analysis

All data were statistically analyzed and plotted using GraphPad Prism (version 9.5) and SPSS software (version 22.0). *T*‐test and one‐way ANOVA were used for comparisons between two and more than three groups of data, respectively, and LSD was used for post‐hoc multiple testing. A *p*‐value less than 0.05 indicates a statistical difference.

## 3 RESULTS

### 3.1 PF reduces the symptoms of CDiph‐induced STC rats

**FIGURE 1:**
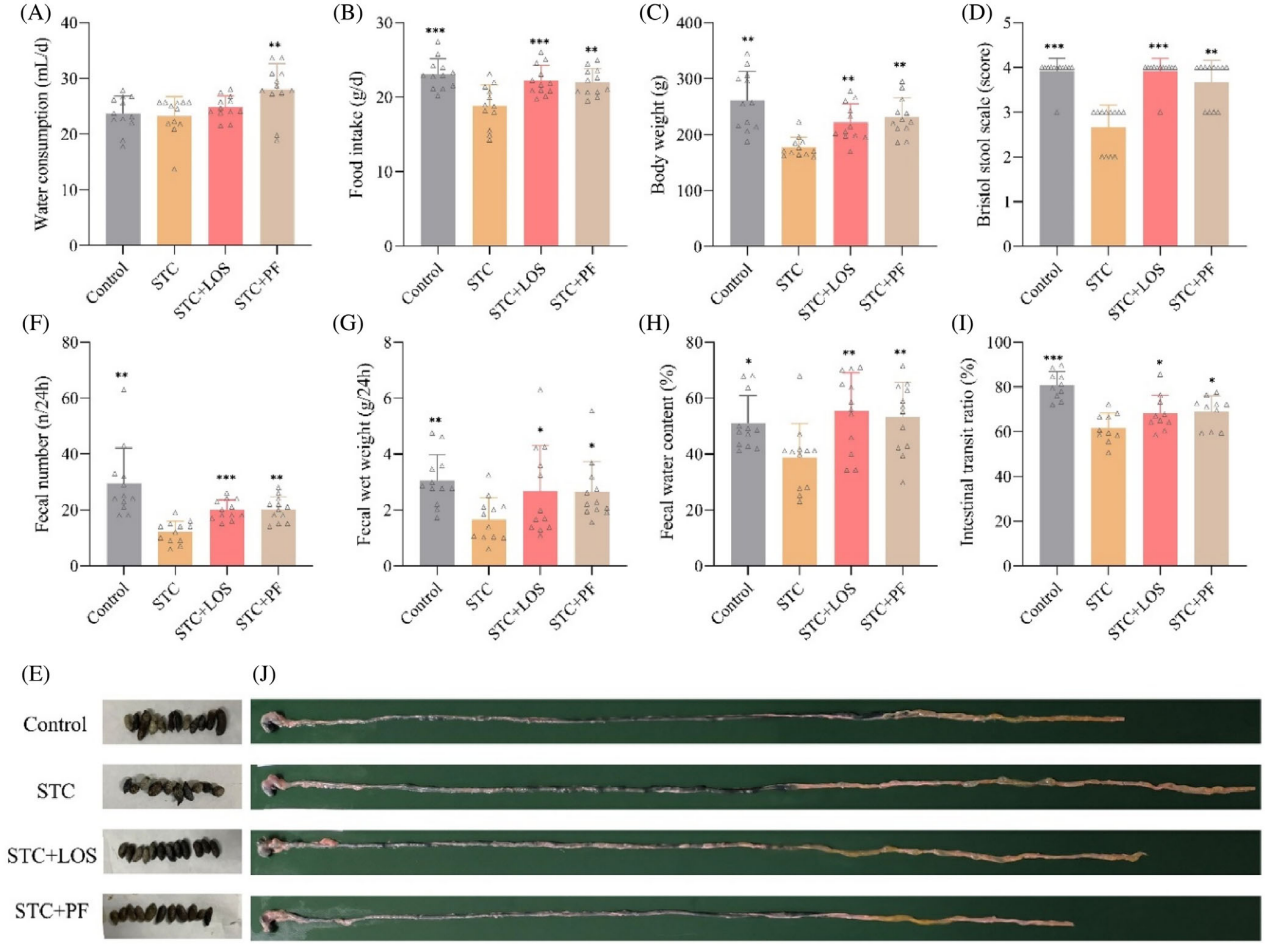
Effect of PF on the general condition, defecation, and intestinal propulsion rates in CDiph‐induced STC rats. (A) Daily water consumption of rats in each group during treatment. (B) Daily food intake of rats in each group during treatment. (C) Body weight of rats at the end of treatment. (D) Bristol score of fecal characteristics of rats at the end of treatment. (E) Physical figures of feces of rats in each group at the end of treatment. (F) 24‐h fecal pellet count of rats at the end of the intervention. (G) 24‐h WW of rat feces at the end of the intervention. (H) At the end of treatment, water content of rat feces (24‐h feces). (I) Small intestinal transit ratio of rats in each group at the end of the intervention. (J) Typical figures of charcoal powder propulsion distance in rats' small intestines. Differences compared to the STC group; *p < 0.05, **p < 0.01, ***p < 0.001. CDiph, compound diphenoxylate; PF, paeoniflorin; STC, slow transit constipation.

Drinking water consumption in the PF group was significantly higher than in the STC group, but there were no significant differences between the other groups (Figure 1A). Food intake and body weight loss in STC rats were significantly improved by PF and LOS treatments (Figure 1B,C). Fecal scores, 24‐h fecal pellet counts, fecal WW, fecal water content, and small intestinal transit ratio were significantly reduced in model rats, whereas they were remarkably increased after PF and LOS interventions (Figure 1D–J). In summary, PF not only improved the general condition of STC rats but also wetted feces, increased fecal volume, accelerated colon transit, and relieved constipated symptoms.

### 3.2 PF mitigates CDiph‐induced colonic pathological injury in STC rats

**FIGURE 2:**
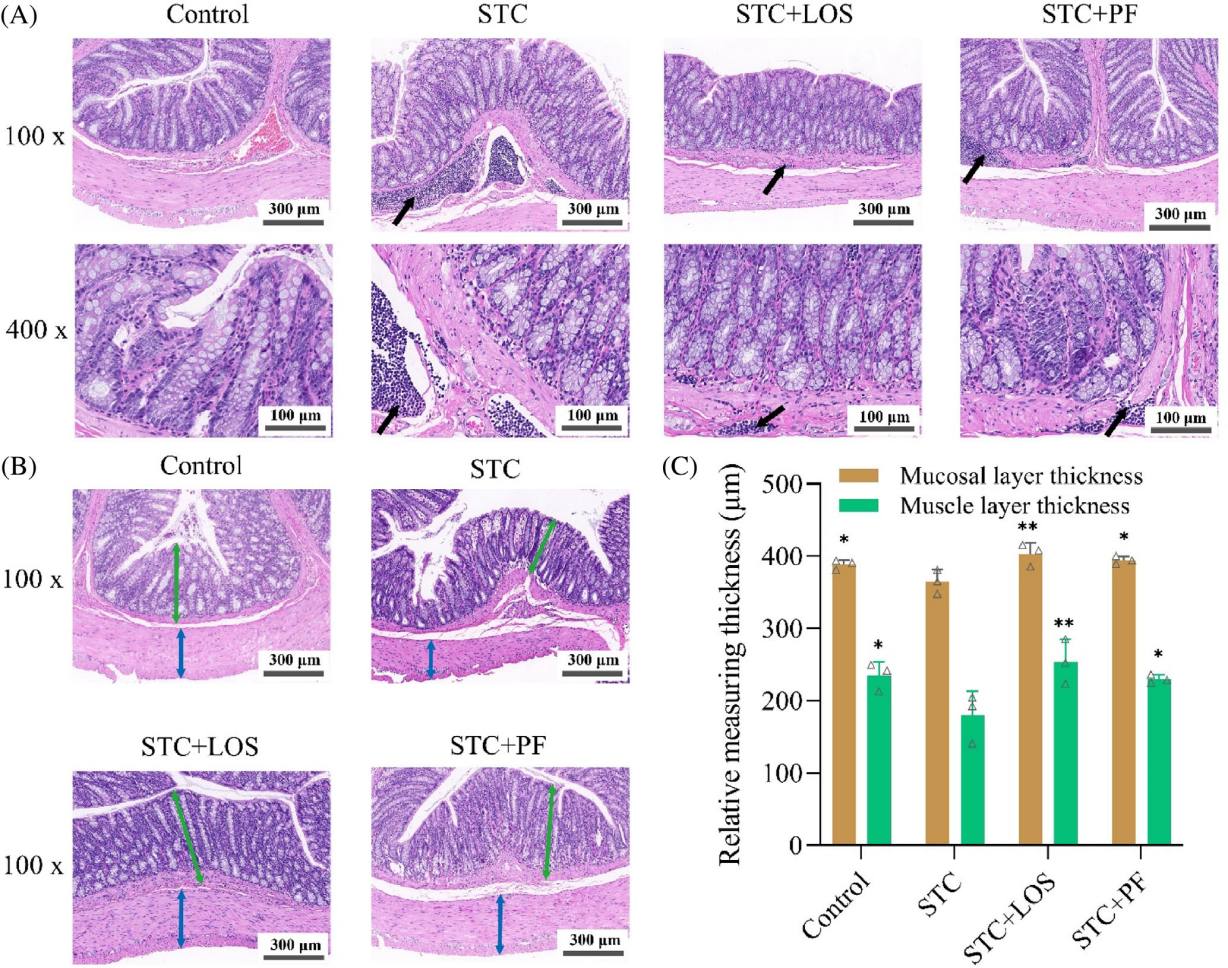
Effect of PF on the morphology of colonic tissue in CDiph‐induced STC rats. (A) Pathological morphology of HE staining of rat colon tissues in each group (magnification 100×, scale bar = 300 μm; magnification 400×, scale bar = 100 μm, black arrows indicate lymphocyte). (B) Typical diagrams of HE‐stained mucosal and muscular layer thicknesses of rat colon tissues in each group (magnification 100×, scale bar = 300 μm, green bi‐directional arrows indicate mucosal layer thickness and blue bi‐directional arrows indicate muscular layer thickness). (C) Measured values of mucosal and muscular layer thicknesses of rat colon tissues in each group. Differences compared to the STC group; *p < 0.05, **p < 0.01. CDiph, compound diphenoxylate; PF, paeoniflorin; STC, slow transit constipation.

As shown in Figure 2A–C, the morphology of colonic tissue under the light microscope in the control group was normal. In the STC group, the colonic mucosa was mildly edematous, the number of colonic glands was reduced, inflammatory cell infiltration could be seen in the lamina propria and submucosa of the mucosa, and the colonic mucosal layer and muscle layer were markedly thinned. Whereas the above pathological injuries were attenuated after treatment with PF and LOS. In conclusion, PF was able to repair the pathological damage of the rat colon induced by CDiph to a certain extent.

### 3.3 PF attenuates CDiph‐induced abnormal intestinal fluid metabolism in STC rats

**FIGURE 3:**
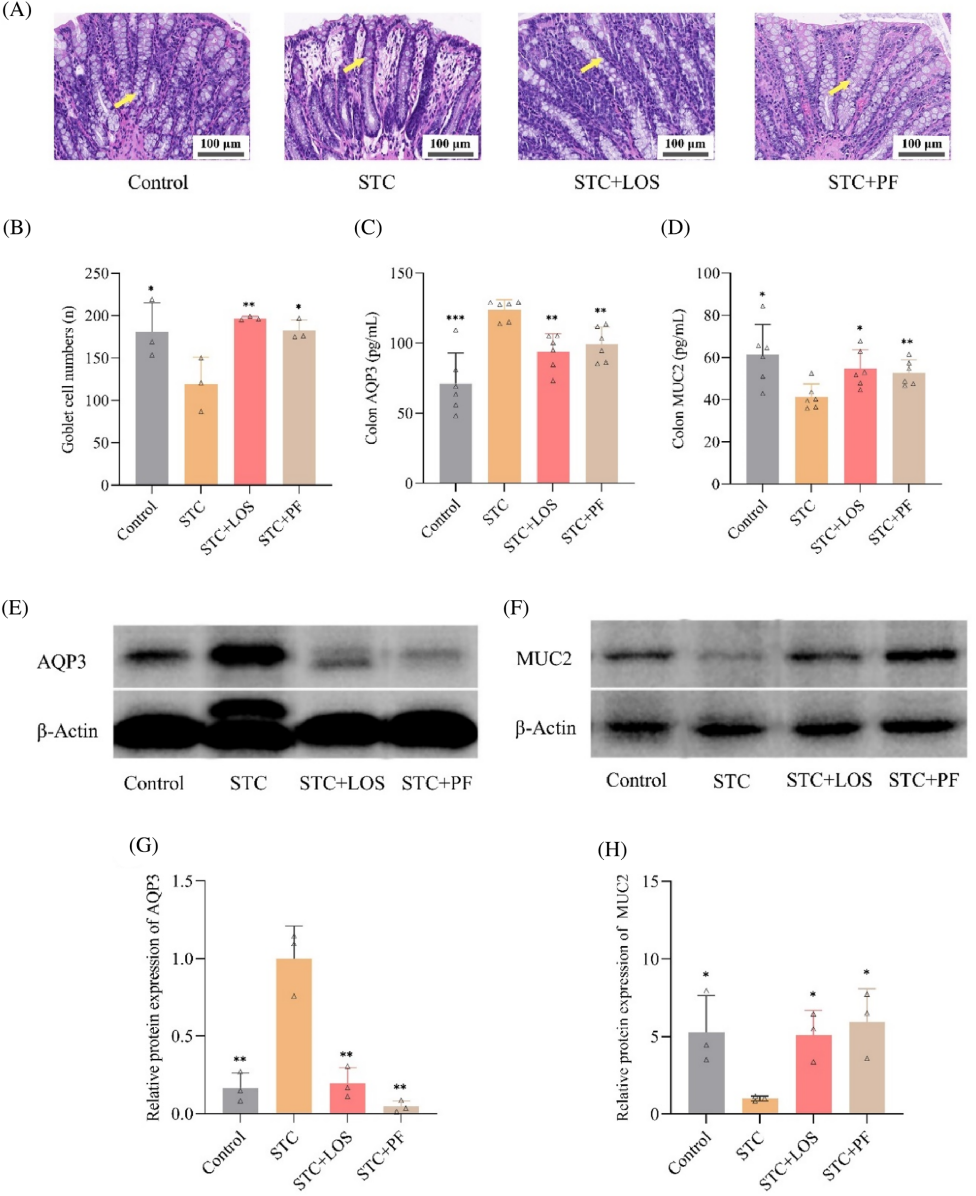
Effect of PF on colonic fluid metabolism in CDiph‐induced STC rats. (A) Typical diagram of HE‐stained vacuolated goblet cells in rat colon tissue (magnification 400×, scale bar = 100 μm, yellow arrows indicate goblet cells). (B) Count of HE‐stained goblet cells in rat colon tissue. (C) ELISA results of AQP3 protein expression level in rat colon tissue. (D) ELISA results of MUC2 protein expression level in rat colon tissues. (E) Gel representative graph of AQP3 protein expression level in rat colon tissues. (F) Gel representative graph of MUC2 protein expression level in rat colon tissues. (G) Grayscale value statistical graph of AQP3 protein expression in rat colon tissues. (H) Statistical graph of grayscale values of MUC2 protein expression in rat colon tissues. Differences compared to the STC group; *p < 0.05, **p < 0.01, ***p < 0.001. CDiph, compound diphenoxylate; ELISA, enzyme‐linked immunosorbent assay; PF, paeoniflorin; STC, slow transit constipation.

We explored the effects of PF on STC colonic fluid metabolism at the tissue and molecular levels the reduced number of goblet cells in the colon of STC rats was significantly restored by treatment with PF and LOS (Figure 3A,B). ELISA and WB results showed that colonic MUC2 protein expression was significantly reduced compared to the control group, and AQP3 protein expression was significantly increased in the STC group. These changes were effectively reversed with PF and LOS (Figure 3C–F). Overall, PF significantly ameliorated colonic fluid metabolism disorders in STC rats.

### 3.4 PF alleviates CDiph‐induced decrease in visceral sensitivity in STC rats

**FIGURE 4:**
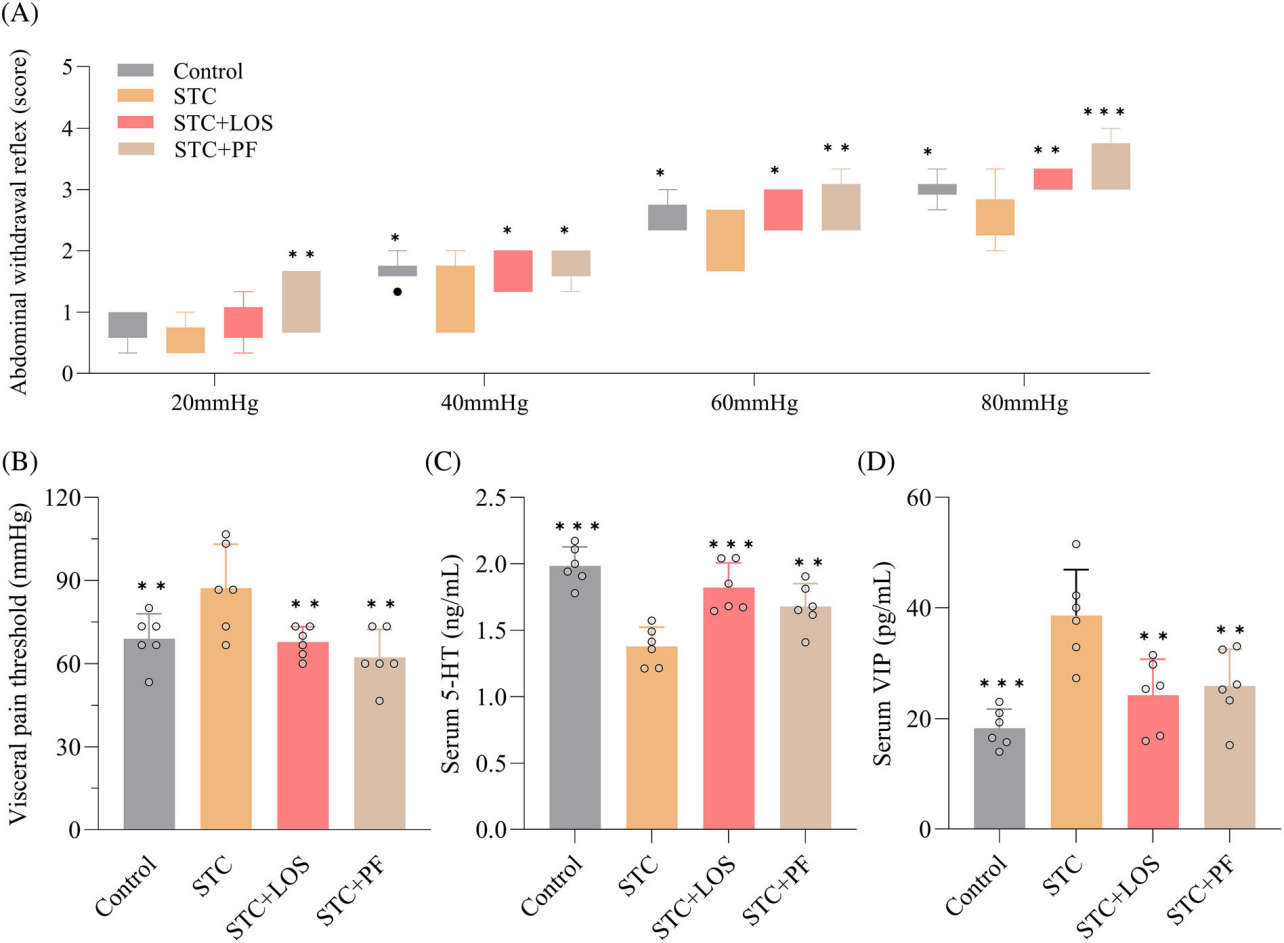
Effect of PF on visceral sensitization in CDiph‐induced STC rats. (A) AWR scores of rats at the end of treatment. (B) Visceral pain thresholds of rats at the end of the intervention. (C) Serum 5‐HT levels in rats. (D) Serum VIP levels in rats. Differences compared to the STC group; *p < 0.05, **p < 0.01, ***p < 0.001. AWR, abdominal withdrawal reflex; CDiph, compound diphenoxylate; PF, paeoniflorin; STC, slow transit constipation; VIP, vasoactive intestinal peptide.

We investigated the effects of PF on visceral sensitivity in STC rats using behavioral and immunological methods. As shown in Figure 4A, the AWR scores of STC rats were significantly lower than those of the control group, except for 20 mmHg pressure, and the PF group scores were considerably higher than those of the STC group at all pressures. In addition, PF and LOS treatment significantly reduced the elevated internal pain threshold of STC rats (Figure 4B). ELISA results showed that serum 5‐HT levels decreased and VIP levels increased in the STC group compared with the control group, and these changes were significantly reversed by PF and LOS treatment (Figure 4C,D). Briefly, PF enhanced the visceral sensitivity of STC rats.

### 3.5 PF activates the ASIC3/ERK pathway in CDiph‐induced STC rats

**FIGURE 5:**
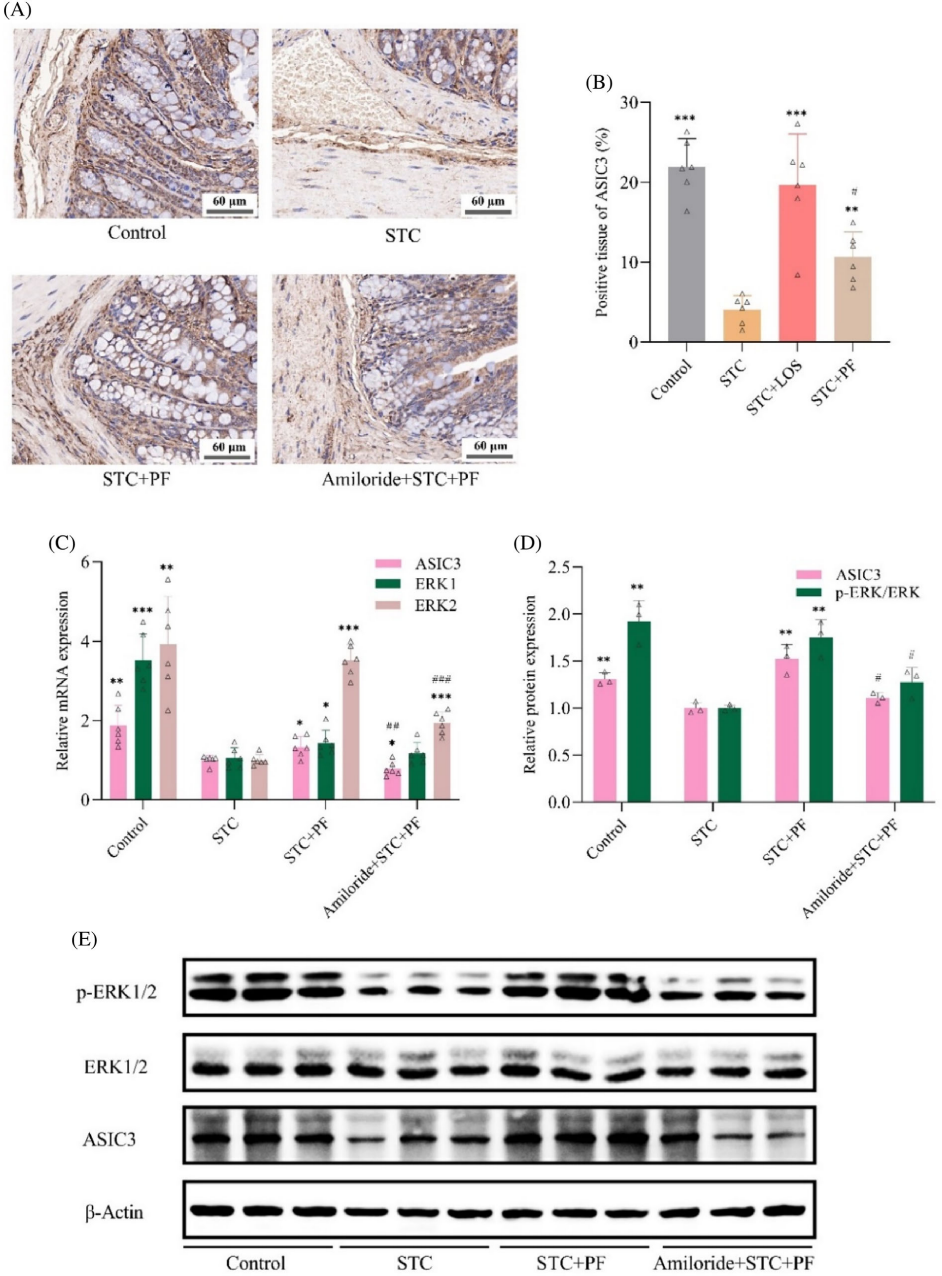
Effect of PF on the ASIC3/ERK signaling pathway in the colon of CDiph‐induced STC rats. (A) Representative immunohistochemical map of ASIC3 protein expression in rat colon tissue (magnification 400×, scale bar = 60 μm). (B) Percentage of positive area for ASIC3 protein expression in rat colon tissues. (C) Transcription levels of ASIC3, ERK1, and ERK2 genes in rat colon tissues. (D) A grayscale value of protein expression of ASIC3 and the ratio of (p‐ERK1/2)/(ERK1/2) protein grayscale value in rat colon tissues. (E) Gel representative plots of ASIC3, ERK1/2, and p‐ERK1/2 protein expression levels in rat colon tissues. Differences compared to the STC group; *p < 0.05, **p < 0.01, ***p < 0.001. Differences between the amiloride group compared to the PF group; #*p* < 0.05, ##*p* < 0.01, ###*p* < 0.001. ASIC3/ERK, acid‐sensitive ion channel 3/extracellular signal‐regulated kinase; CDiph, compound diphenoxylate; PF, paeoniflorin; STC, slow transit constipation.

We first examined the expression of ASIC3 and ERK in the colon of STC rats and then explored the possibility of ASIC3/ERK as a signaling pathway mediating the laxative effects of PF. As shown in Figure 5A–E, ASIC3 was mainly expressed in the cytoplasm of the submucosa and muscularis of the colon (positive expression in brownish‐yellow color). The gene transcription, protein expression, and positive expression area of colonic ASIC3 in STC rats were significantly lower compared with the control group; in addition, the transcription levels of colonic ERK1 and ERK2 genes and the ratio of colonic p‐ERK1/2 expression level to total ERK1/2 protein expression level were markedly less than those of the control group. PF treatment significantly increased the expression levels of the above indicators compared with the STC group. By contrast, rats injected with amiloride (an ASIC3 inhibitor) showed a significant decrease in the expression levels of the above indicators compared with the PF group, except for the ERK1 gene transcript level. In conclusion, PF alleviated the downregulation of ASIC3/ERK‐related gene transcription and protein expression in the colon of STC rats. However, this effect was inhibited by amiloride. Thus, PF activated the colonic ASIC3/ERK signaling pathway in STC rats.

### 3.6 PF reverses CDiph‐induced abnormalities of intestinal fluid metabolism in STC rats through activation of the ASIC3/ERK pathway

**FIGURE 6:**
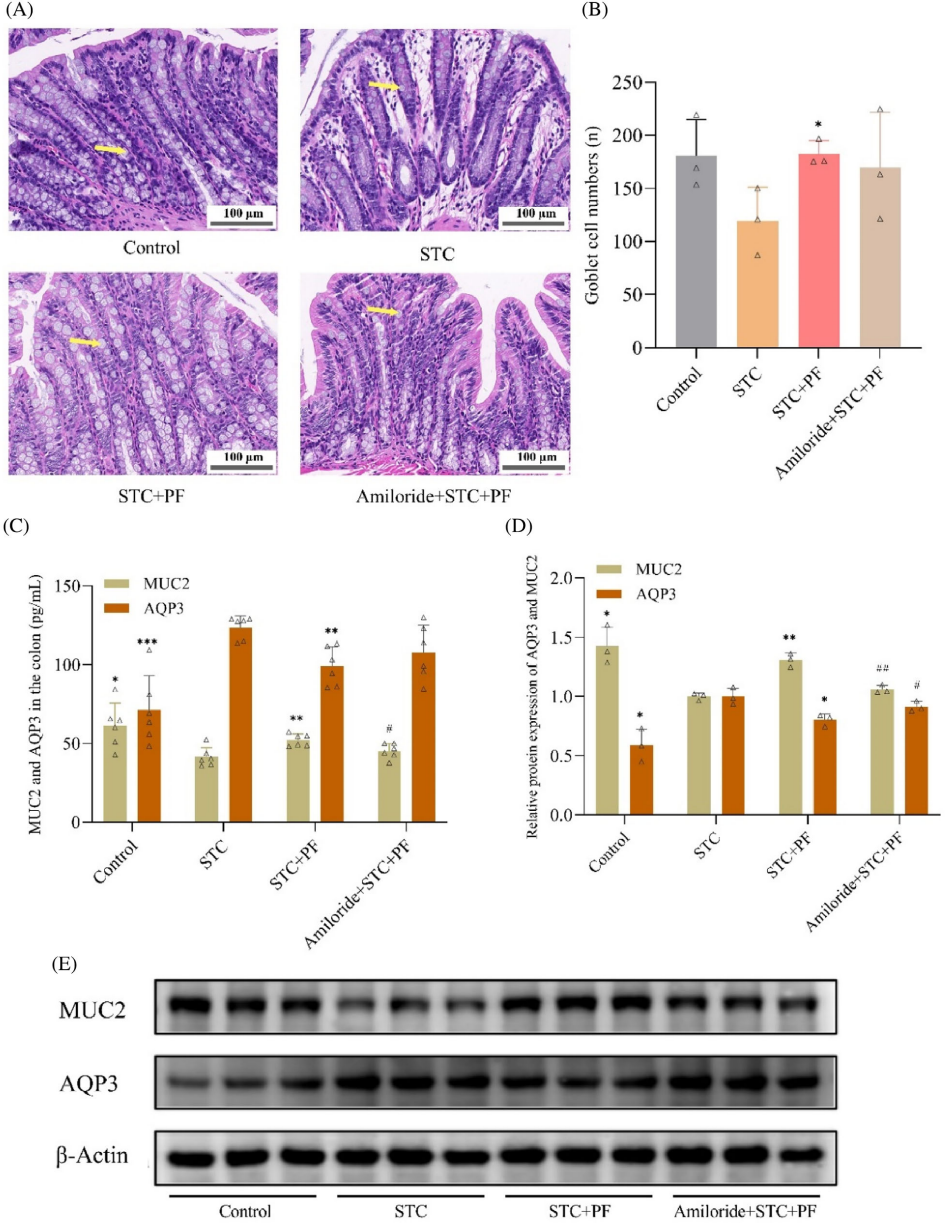
PF alleviates CDiph‐induced colonic fluid metabolism disorder in STC rats by activating the ASIC3/ERK pathway. (A) Typical diagram of HE‐stained vacuolated goblet cells in rat colon tissue (magnification 400×, scale bar = 100 μm, yellow arrows indicate goblet cells). (B) Count of HE‐stained goblet cells in rat colon tissue. (C) Statistical graph of ELISA results of MUC2 and AQP3 expression levels in rat colon tissues. (D) Statistical graph of the grayscale values of MUC2 and AQP3 protein expression in rat colon tissues. (E) Gel representative graph of MUC2 and AQP3 protein expression levels in rat colon tissues. Differences compared to the STC group; *p < 0.05, **p < 0.01, ***p < 0.001. Differences between the amiloride group compared to the PF group; #*p* < 0.05, ##*p* < 0.01. ASIC3/ERK, acid‐sensitive ion channel 3/extracellular signal‐regulated kinase; CDiph, compound diphenoxylate; ELISA, enzyme‐linked immunosorbent assay; PF, paeoniflorin; STC, slow transit constipation.

To determine whether PF could attenuate the abnormalities of fluid metabolism in STC rats by activating the ASIC3/ERK signaling pathway, we compared the relevant indices in the amiloride group with those in other groups. As shown in Figure 6A,B, PF significantly increased the number of STC colonic goblet cells. While the number of goblet cells in the amiloride group showed a decreasing trend compared to the PF group, it is suggested that the effect of PF on increasing STC colonic goblet cells was partially attenuated by amiloride. Meanwhile, ELISA and WB results also indicated that the impact of PF in upregulating MUC2 protein and downregulating AQP3 protein in STC rat colon was counteracted by amiloride (Figure 6C–E). Taken together, the findings suggest that PF could alleviate intestinal fluid metabolism disorders in STC rats by activating the ASIC3/ERK signaling pathway.

### 3.7 PF ameliorates CDiph‐induced visceral sensitivity in STC rats by activating the ASIC3/ERK pathway

**FIGURE 7:**
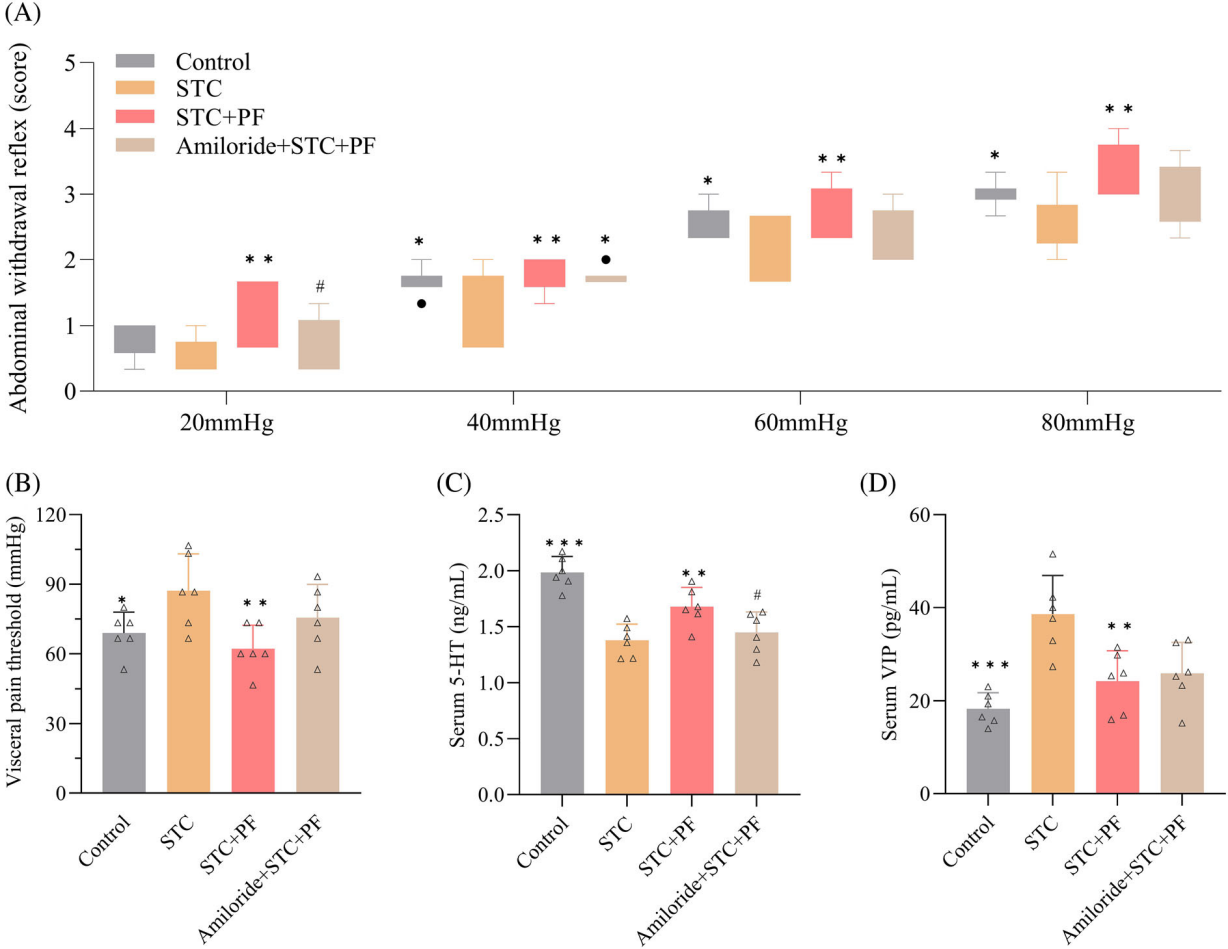
PF ameliorates CDiph‐induced abnormal visceral sensitivity in STC rats by activating the ASIC3/ERK pathway. (A) AWR scores of rats in each group. (B) Visceral pain thresholds of rats in each group. (C) Serum 5‐HT levels of rats in each group. (D) Serum VIP levels of rats in each group. Differences compared to the STC group; *p < 0.05, **p < 0.01, ***p < 0.001. Differences between the amiloride group compared to the PF group; #*p* < 0.05. 5‐HT, 5‐hydroxytryptamine; ASIC3/ERK, acid‐sensitive ion channel 3/extracellular signal‐regulated kinase; AWR, abdominal withdrawal reflex; CDiph, compound diphenoxylate; PF, paeoniflorin; STC, slow transit constipation; VIP, vasoactive intestinal peptide.

To investigate whether the effect of PF on increasing visceral sensitivity in STC rats could be realized by activating the ASIC3/ERK pathway, we observed the differences between the relevant indexes in the amiloride group and the other groups. As shown in Figure 7A–D, amiloride‐treated rats showed a decreasing trend or significant decrease in AWR score, an increasing trend in pain threshold, a significant decline in serum 5‐HT, and an increasing trend in serum VIP compared with the PF group, whereas there were no significant differences compared with the STC group in any of the indexes except for the AWR score at 40 mmHg. This suggests that the effect of PF in increasing visceral sensitivity in STC rats was partially attenuated by amiloride. In summary, PF can improve visceral sensitivity in STC rats by activating the ASIC3/ERK pathway.

## 4 DISCUSSION

STC is a common clinical health problem, and its therapeutic agents and mechanisms of action have received widespread attention from researchers. CDiph is commonly used to induce STC animal models because of its inhibitory effect on intestinal motility and is more likely to cause constipation than loperamide.^22^ We first constructed a rat model of STC by referring to the literature method,^20^ and then treated them with the optimal laxative dose of PF (40 mg/kg/day) screened in a previous study.^7^ At the same time, lactulose oral solution was used as a positive control drug to ensure the reliability of the evaluation of its efficacy.^22^ After treatment, PF effectively alleviated constipation symptoms and reduced histopathological damage to the colon in STC rats.

Disturbances in colonic fluid absorption and secretion are involved in the pathogenesis of STC,^3^ and restoration of colonic fluid balance is significant in its treatment.^1^ MUCs and AQPs play essential roles in colonic fluid secretion and transport. The amount of MUC determines the lubricity of mucus in the intestine, and the efficiency of food residue transport in the intestine increases with higher mucus lubricity.^10^ MUC2, secreted by goblet cells, is the major gel MUC involved in the composition of the intestinal mucus layer,^23^ and its expression is reduced in the intestines of the STC model.^24^ AQPs control water entry and exit through channels formed in the membranes of colonic epithelial cells.^25^ AQP3 is abundantly present in the colon, and its elevated expression increases water reabsorption, thereby causing constipation.^9^, ^26^ Drugs with PF as the main ingredient have been reported to have regulatory effects on MUC2 and AQP3. For example, Painong San, a Chinese herbal compound for the treatment of colitis, increased the expression of MUC2 protein in the colon,^27^ and *P. lactiflora* downregulated the expression level of AQP3 in the colon of constipated rats.^28^ Our study also showed that PF significantly increased MUC2 protein expression and the number of colonic goblet cells and decreased AQP3 protein expression in CDiph‐induced STC rats, thereby improving intestinal fluid metabolism disorders.

Visceral sensitivity refers to the sensitivity of visceral receptors to physiological stimuli and their responsiveness to injurious stimuli and is closely related to intestinal motility.^29^ Both patients and models of FC exhibit reduced visceral sensitivity.^1^, ^11^, ^30^ STC patients require higher than normal intraluminal pressure to induce defecation.^9^ Recently, in addition to AWR scores, several neurotransmitters have been found to respond to changes in visceral sensitivity. For example, resveratrol and the Chinese herbal compound Chang‐Kang‐Fang were reported to reduce colonic 5‐HT and VIP levels, respectively, while decreasing visceral sensitivity in IBS rats.^31^, ^32^ Therefore, we used 5‐HT and VIP as objective indicators to assist in evaluating visceral sensitivity in our experiments. PF was reported to reduce AWR scores and upregulate colonic VIP and 5‐HT levels in IBS mice.^33^ It is evident that PF has a modulatory effect on visceral sensation and related factors, but whether this is connected to its laxative effect has rarely been examined. The STC rats in our experiments had reduced visceral sensitization, as evidenced by lower AWR scores, higher visceral pain thresholds, lower serum 5‐HT levels, and higher VIP levels. All of these changes were effectively reversed after treatment with PF. Thus, both subjective and objective indicators confirm that PF increases visceral sensitization in STC rats, thereby increasing the body's perception of intestinal contents needed to induce the defecation reflex.

ASICs are members of the amiloride‐sensitive epithelial sodium channel/degenerate protein superfamily and play an important role in regulating gastrointestinal mechanosensitivity.^34^ ASIC3, the most commonly expressed isoform of the ASIC family in the colon, is a target for controlling visceral pain.^13^ Intestinal ASIC3 expression was reported to be significantly reduced in rats transplanted with fecal bacteria from children with FC, with reduced visceral sensitivity and prolonged colonic transit time.^30^ ASICs are also involved in the regulation of MUC5B secretion by airway acidification.^35^ PF was reported to inhibit ASICs from exerting cerebroprotective effects on Parkinson's model rats.^6^ Therefore, we used ASIC3 as a breakthrough point to investigate the mechanisms by which PF improves visceral sensation and fluid metabolism in STC, and we could not help but consider how ASIC3 functions. A hybrid kinase involved in cell survival, ERK, came into view. It was shown that activation of ERK is not only engaged in enhancing and maintaining visceral sensitivity,^36^ but is also associated with increased intestinal MUC2 expression in colitis mice by allyl isothiocyanate,^37^ and accompanied by downregulation of colonic AQP3 in an IBS model.^17^ PF was found to activate the ERK‐CREB signaling pathway exerting a cerebroprotective effect on chronically stressed rats.^19^ Thus, ERK may also be associated with PF regulation of water balance and sensitivity in the gut of STC rats. While there appears to be a regulatory relationship between ASIC3 and ERK, it has been found that hypoxia and ASIC3 overexpression activate the MAPK pathway (upregulation of p‐ERK1/2 and p‐MAPK expression), thus affecting the behavior of myeloid cells.^38^

Still, this regulatory relationship has not been reported in constipation. Our study found that gene transcription of ASIC3 and ERK (ERK1 and ERK2), protein expression of ASIC3, and (p‐ERK1/2)/(ERK1/2) ratios were reduced in the colon of STC rats, and these metrics were upregulated after using PF. We also found that in rats treated with PF in combination with amiloride, a broad‐spectrum inhibitor of ASICs that has a blocking effect on ASIC3,^39^ the effects of PF on upregulating ERK2 gene transcription and activating p‐ERK1/2 were counteracted, in addition to the inhibition of ASIC3. Meanwhile, amiloride differentially attenuated the effects of PF in regulating intestinal fluid and sensitivity in STC rats. Notably, ERK gene transcript levels differed between groups. Notably, ERK gene transcript levels differed between groups. Compared with the PF group, treatment with the ASIC3 inhibitor amiloride only reduced the transcript levels of the ERK2 gene in rats and had no significant effect on the transcript levels of the ERK1 gene, but it still reduced the ratio of p‐ERK1/2 proteins to total ERK1/2 proteins (a reduction in this ratio represents the inhibition of p‐ERK1/2). These results indicate that ASIC3 may achieve regulation of ERK1/2 phosphorylation mainly by affecting ERK2. Our conjecture seems to be supported by the results of previous investigators, who found that ERK1^−/−^ mice survived and developed normally, whereas ERK2 null mutations lead to the embryonic lethality of mice, suggesting that ERK2 may compensate for the consequences of ERK1 deficiency.^40^ However, more in‐depth studies are needed to confirm this. Accordingly, we tentatively conclude that PF ameliorates the disturbed colonic fluid metabolism and abnormal visceral sensitivity in STC rats by activating the ASIC3/ERK signaling pathway.

In conclusion, this study has explored the laxative mechanism of PF based on previous studies and found that PF affects colonic fluid metabolism and visceral sensitivity in STC rats by regulating the ASIC3/ERK signaling pathway, attenuates colonic pathological injury, and alleviates constipation symptoms. Our findings partly enhance the comprehension of PF's pharmacological mechanism in treating STC and potentially provide novel ideas for developing therapeutic medications for STC. However, like all research, the study does have some limitations. On the one hand, the mechanisms regulating intestinal fluid metabolism and visceral sensitivity are very complex. Although we have investigated representative proteins and neurotransmitters, more molecules need to be designed for exploration. On the other, since the ASIC3/ERK signaling pathway is seldom mentioned in constipation, although we have initially clarified the role of this pathway in the laxative effect of PF with an ASIC3 inhibitor, further studies are needed to determine its regulatory mechanism. In the future, we can conduct in‐depth investigations using cellular experiments, gene editing, and other technologies to enhance the depth and breadth of our findings.

## CONFLICT OF INTEREST STATEMENT

The authors declare no conflicts of interest.
